# Mitochondria Change Dynamics and Morphology during Grapevine Leaf Senescence

**DOI:** 10.1371/journal.pone.0102012

**Published:** 2014-07-10

**Authors:** Cristina Ruberti, Elisabetta Barizza, Martina Bodner, Nicoletta La Rocca, Roberto De Michele, Francesco Carimi, Fiorella Lo Schiavo, Michela Zottini

**Affiliations:** 1 Dipartimento di Biologia, Università degli Studi di Padova, Padova, Italy; 2 Istituto di Bioscienze e Biorisorse, Consiglio Nazionale delle Ricerche (CNR-IBBR), Corso Calatafimi, Palermo, Italy; Iowa State University, United States of America

## Abstract

Leaf senescence is the last stage of development of an organ and is aimed to its ordered disassembly and nutrient reallocation. Whereas chlorophyll gradually degrades during senescence in leaves, mitochondria need to maintain active to sustain the energy demands of senescing cells. Here we analysed the motility and morphology of mitochondria in different stages of senescence in leaves of grapevine (*Vitis vinifera*), by stably expressing a GFP (green fluorescent protein) reporter targeted to these organelles. Results show that mitochondria were less dynamic and markedly changed morphology during senescence, passing from the elongated, branched structures found in mature leaves to enlarged and sparse organelles in senescent leaves. Progression of senescence in leaves was not synchronous, since changes in mitochondria from stomata were delayed. Mitochondrial morphology was also analysed in grapevine cell cultures. Mitochondria from cells at the end of their growth curve resembled those from senescing leaves, suggesting that cell cultures might represent a useful model system for senescence. Additionally, senescence-associated mitochondrial changes were observed in plants treated with high concentrations of cytokinins. Overall, morphology and dynamics of mitochondria might represent a reliable senescence marker for plant cells.

## Introduction

Senescence defines the final stage of development of any organ. In leaves, senescence is particularly spectacular since it is characterized by chlorophyll degradation and the display of other pigments. Senescence is aimed to an ordered disassembly of the cellular components and recycling of nutrients that are exported to other plant organs. Eventually, senescence terminates with a genetically programmed cell death (PCD) [1]. Senescence is a complex physiological process whose onset and regulation is under control of several internal (hormones, plant stage and organ age) and external (light, nutrient availability, biotic and abiotic stresses) stimuli. The complexity of the process is revealed by the change in expression of thousand genes and the activation of many signalling pathways [2].

Since senescence is an active and slow process, cells require energy to stay alive until the onset of PCD. Early in the senescence process, the photosynthetic capacity of leaves drops, due to chlorophyll degradation. The energy demand of senescing cells therefore relies completely on mitochondria [3]. Moreover, besides their central role in energy and carbon metabolism [4], mitochondria appear to play a significant role as stress sensors and signal dispatchers during PCD events. In animals, but also in plants, mitochondria regulate PCD by releasing pro-apoptotic factors such as cytochrome c and possibly also through their dynamics and morphology alterations [5]. Although mitochondria are often portrayed as static, oval or rod-shaped organelles, recent studies have demonstrated that their shape and distribution are variable [6]. Changes in the distribution pattern of mitochondria and their movement appear to be critical in executing their cellular functions. A link between PCD and plant mitochondrial dynamics and morphology alterations has been already reported in protoplasts of *Arabidopsis* [7], during dark-induced senescence in Arabidopsis leaves [3] and in cell cultures of *Medicago truncatula* [8] experiencing starvation or treatment with high levels of cytokinins, treatments able to trigger senescence-like processes [9]–[13]. However, despite their utility as a simplified model, protoplasts and cell cultures might not cover all the aspects of a complex developmental process such as leaf senescence.

Here we investigated whether the shape and dynamics of mitochondria in leaves of grapevine (*Vitis vinifera*) change during senescence. Grapevine is an important crop species and a model for deciduous perennial plants, characterized by a full seasonal senescing syndrome and whose genome has been fully sequenced and annotated [14]–[16]. We also extended our observations to leaves treated with high concentrations of cytokinins and to starved cell cultures, in order to test whether these senescence-like processes resembled spontaneous leaf senescence in terms of mitochondrial modifications.

## Materials and Methods

### Cloning

For the expression of the GFP targeted to mitochondria, the *β-GFP* coding sequence was subcloned from the *β-GFP* plasmid [17], [18] into the pBI121 binary vector (Clontech Laboratories, USA), by replacing the *β*-*glucuronidase* coding sequence into the *BglII/SacI* restriction sites as described by Zottini et al. [19]. The pBI121 binary vector harbouring the *neomycin phosphotransferase II* coding sequence allowed the selection of kanamycin resistant transgenic cells.

Competent cells of *Agrobacterium tumefaciens* GV3101 strain (resistant to rifampicin and gentamycin) were prepared according to Sambrook et al. [20] and the pBI121 *β-GFP* binary vector was introduced by electroporation as reported by Zottini et al. [19]. Bacterial growth was optimized by using YEP medium (10 g/L Bacto-Trypton, 10 g/L yeast extract, 5 g/L NaCl; pH 7.0). The medium was supplemented with appropriate antibiotic selection (100 µg/mL rifampicin, 50 µg/mL gentamycin, 50 µg/mL kanamycin).

### Generation of embryogenic cell lines and transgenic plants selection

Embryogenic cell lines of grapevine (‘Moscato giallo' cultivar) were produced from stigma/style cultures as described by Carimi et al. [21]. Briefly, explants were dissected from unopened flowers and placed on Nitsch and Nitsch medium (NN; [22]) salts and vitamins, 88 mM sucrose, 9 µM BA (6-benzylaminopurine) and 10 µM 2-naphthoxyacetic acid. Medium pH was adjusted to 5.7 before the addition of 8 g/L plant agar (Duchefa) and autoclaved at 121°C for 20 min. Cultures were placed in an acclimatized cabinet at 25±1°C and 16 h light photoperiod, and subcultured at 30-day intervals. White embryogenic globules, around 1–3 mm in size, were separated from the callus grown from the original stigma/style explants and were cultured alternating, every 3 weeks, solid Murashige and Skoog [23] growth regulator free medium (MS-) and solid (8 g/L plant agar, Duchefa) Gamborg B5 medium (B5, Duchefa, Netherlands) [24] supplemented with 2.26 µM 2,4-dichlorophenoxy-acetic acid (2,4-D, SIGMA) (B5F medium).

The transformation procedure was performed as described below. Cell suspensions were initiated from habituated embryogenic cultures by transferring 1 g of pre-embryogenic masses collected from solid MS- to Erlenmeyer flasks (250 mL) filled with 50 mL liquid MS- medium. The flasks were cultured for 3 days on an orbital shaker at 80 rpm and incubated at 25°C in the dark. Before transformation with *A. tumefaciens*, 500 mg of pre-embryogenic masses and embryos were transferred into a Petri dish contained 1 mL of induction liquid medium (ILM, i.e. NN medium supplemented with 58 mM sucrose and 2.26 µM 2,4-D) and incised with a sharp razor blade.

Bacteria suspension was prepared as in Zottini et al. [19]. *A. tumefaciens* suspension was diluted to OD_550_ 0.5 in ILM and added to the dissected embryos previously transferred to bacteria-free ILM. Embryos were incubated at room temperature in the dark for 10 min, after which the cultures were washed 5 times with ILM (3 min each). Infected embryos were blotted dry on sterile filter paper and then plated on NN solid medium and incubated at 25°C in the dark. Two days later, the cultures were transferred to NN solid medium supplemented with 300 µg/mL cefotaxime and maintained at 25°C in the dark. After 10 days the cultures were transferred on NN solid medium supplemented with 20 µg/mL kanamycin and 300 µg/mL cefotaxime. After 20 days the cultures were transferred on NN solid medium supplemented with specific antibiotics (40 µg/mL kanamycin and 300 µg/mL cefotaxime) and subcultured at 20 day-intervals. Embryo clusters were collected and transferred to NN hormone free solid medium for germination. Each somatic embryo was transferred to Microbox Containers (Duchefa) on solid (8 g/L plant agar, Duchefa) half strength MS medium, supplemented with 44 mM sucrose. Plantlets were then multiplied by clonal propagation. All plant material was maintained in a growth chamber at 25±1°C under a 16 h light photoperiod, and a photosynthetic photon flux of 35 µmol m^−2^ s^−1^ Osram cool-white 18 W fluorescent lamps. GFP-fluorescence of *β-GFP* stable transformed grapevine plants was analysed in leaves using a stereomicroscope (Leica MZ16 F) equipped with white and UV light.

### Hydroponic cultivation

Transformed plants were obtained *in vitro* and then transferred to hydroponic culture conditions. The nutrient solution was optimised for *V. vinifera* plants: 0.5 mM KH_2_PO_4_, 0.5 mM K_2_SO_4_, 2 mM Ca(NO_3_)_2_×4H_2_O, 0.65 mM MgSO_4_, 0.5 µM H_3_BO_3_, 0.045 µM CuSO_4_×5H_2_O, 0.05 µM ZnSO_4_×7H_2_O, 0.02 µM (NH_4_)_6_Mo_7_O_24_×4H_2_O, 0.5 µM MnSO_4_, 10 µM Fe-EDDHA. Under hydroponic conditions, plants were grown in polypropylene culture vessels (Duchefa) containing a floating polystyrene ring used as a plant support.

### Cytokinin treatments

Stem cuttings, containing an internode, a node and a still expanding leaf, were dissected from hydroponic grown plants. Stem cuttings were transferred into plastic tubes containing 6 mL hydroponic solution and maintained in a climate growth chamber at 25°C under a 16 h light photoperiod. Leaves were analysed 12 days after treatment with 100 µM BA.

### Grapevine suspension cell cultures

Establishment and maintenance of suspension cell cultures are described in Zottini et al. [19]. Briefly, grapevine cell lines were obtained from leaf disk explants incubated in solid B5F medium. After several subculture cycles, aliquots of callus were inoculated into liquid B5F medium. Every week, 2 mL of suspension cell cultures were transferred to Erlenmeyer flasks (250 mL) filled with 50 mL liquid B5F medium. The suspension cultures were maintained in a climate growth chamber at 25±1°C on an orbital shaker (80 rpm) under a long photoperiod (16 h light and 8 h dark). To determine fresh weight, intact cells were separated from culture medium and cell debris through a vacuum filtration unit (Sartorius, Florence, Italy).

### DNA analysis

DNA was isolated from *V. vinifera* cell cultures as described in [9]. For DNA fragmentation analysis, 10 µg of each sample DNA were electrophoresed on a 1% (w/v) agarose gel containing TAE buffer (40 mM Tris–acetate, 1 mM EDTA) and stained with ethidium bromide.

### Analyses of chlorophyll content and photosynthetic efficiency

Chlorophylls were extracted from leaf tissues by N,N-dimethylformamide and analysed in a double-beam spectrophotometer (GBC UV/VIS 918). Pigment concentrations were calculated using the extinction coefficients of Porra et al. [25].

*In vivo*, chlorophyll fluorescence was measured at room temperature with a Dual PAM-100 (Walz) fluorometer, with a saturating light at 3000 µE m^−2^ s^−1^. Before measurements, plants were dark-adapted for 40 min at room temperature. The parameter Fv/Fm was calculated as the variable (Fm-Fo) over the maximum fluorescence (Fm) [26]. Measurements were replicated at least 3 times.

### Analyses of mitochondrial morphology, volume and number in leaves

Transformed leaves were analysed using a Nikon PCM2000 laser scanning confocal imaging system. For GFP detection, excitation was at 488 nm and emission between 515/530 nm. For the quantification of the number and volume of mitochondria, confocal Z-sections along the leaf thickness were acquired. These analyses were performed on the leaf tips at the different senescence stages (M mature leaves; S1, S2, S3 subsequent senescence leaf stages; 10 replicates for each stage). Image analysis was performed by ImageJ bundle software (http://rsb.info.nih.gov/ij/). Three-dimensional reconstructions of confocal microscope laser scanning images of leaf tissue were obtained by the Volocity 3D Image Analysis Software (PerkinElmer, UK).

### Mitochondria tracking

Time-lapse images of leaf tissues expressing the mitochondria-targeted GFP were acquired every 1.6 s. Size, shape, and intensity parameters were specified so that most organelles would be defined by the software. Tracks combining 12–20 sequential images were calculated by the shortest path model, and mean velocity was calculated for each track. Organelles were tracked using the Volocity 6.0 (PerkinElmer, UK) track utility, and the velocity was calculated for 100–300 organelles/experiment. At least three biological replicates were analysed.

### Semi-quantitative RT-PCR analysis

Total RNA was extracted from entire leaves at different senescence stages (M, S1, S2 and S3), and cultured cells at 7 and 18 days from subculture. Four biological replicates, each with five technical replicates, were considered. RNA isolation was carried out using the “Master Pure Plant RNA Purification” Kit (EPICENTRE Biotechnologies), according to manufacturer's instructions. First strand cDNA synthesis was carried out starting from 2 µg of total RNA, according to the manufacturer's instructions (ImProm Reverse Transcriptase, Promega). Samples were then diluted five folds and used as templates for semi-quantitative RT-PCR. RT-PCR reactions were performed using GoTaq DNA Polymerase (Promega), in a total reaction volume of 50 µL, according to manufacturer's recommendations containing 5 µL diluted cDNA. A PCR amplification cycle was performed using a Hybaid PCR express thermal cycler (VWR, Radnor, Pennsylvania, USA) with an initial denaturation step at 94°C for 2 min, followed by 32 cycles for *VvActin-1* (XM_002282480), 28 cycles for *VvGPDH* (glyceraldehyde-3-phosphate dehydrogenase; XM_002263109), *VvSAG12* and *VvSAG13* (senescence-associated gene 12 and 13; XM_002284937 and XM_002277823, respectively), and 31 cycles for *VvNAM* (no apical meristem family gene; XM_002284618) of 95°C for 20 s, 61°C for 30 s, 72°C for 30 s, and finally with an elongation step at 72°C for 5 min. RT-PCR analyses were performed using the following specific primers: for the *VvActin-1* housekeeping gene 5′-GACAATGGAACTGGAATGGTGAAG-3′ (forward) and 5'-TACGCCCACTGGCATATAGAGAAA-3′ (reverse); for the *VvGPDH* housekeeping gene 5′-CAGGATGCCATGTGGACAA-3′ (forward) and 5'-GTGTTGCCTTCATTGAATGG -3′ (reverse); for *VvSAG12* 5′-AGCTTCCGATGGCAGATG-3′ (forward) and 5′-TCTTCACAGCAGGTGGCA-3′ (reverse); for *VvSAG13* 5′-GCTTCCTGCTCCAGATGC-3′ (forward) and 5′-TGCCACCGTACACACCTG-3′ (reverse); for *VvNAM* 5′-ATGCTCACAATCCGTAACCG-3′ (forward) and 5′-CAGCCACAACATCAAGCATC-3′ (reverse). PCR products were visualised on 1% (w/v) agarose gel containing ethidium bromide [20], and image densitometry analyses were performed using QUANTITY ONE software (Bio-Rad). The expression levels of each gene in leaves and cultured cells were normalised to the expression level of the housekeeping genes *VvActin-1* or *VvGPDH*, respectively. For each experiment five technical replicates were done.

### Analysis of mitochondrial morphology in cell culture

A Nikon PCM2000 laser scanning confocal microscope was used to analyse mitochondrial morphology. The tetramethylrhodamine methyl ester (TMRM) dye (Molecular Probes, Leiden, the Netherlands), a mitochondrial membrane potential sensor, was used to visualise mitochondria in cell cultures as described by Zottini et al. [8]. Cell suspensions (300 µL) were collected at different times during their growth cycle, and incubated for 15 min on a rotary shaker in 700 µL B5F medium containing 1 µM TMRM. Cells were centrifuged for 3 min at 10,000×*g*, the supernatant was discarded and the pellet washed twice with 700 µL B5F. Cells were then resuspended in 500 µL B5F. For microscopy analysis, 100 µL cell suspensions were placed on a microscope slide and visualised under a confocal microscope (excitation 548 nm, emission 573 nm). Images were processed using ImageJ bundle software (http://rsb.info.nih.gov/ij/). For mitochondrial morphology experiments, a randomized complete block design was used with three replicates (individual Erlenmeyer flasks). Each experiment was repeated three times.

### Cell viability assay

Cell viability was determined by the fluorescein diacetate (FDA, SIGMA) method according to Amano et al. [27]. Immediately before each assay, a stock solution of FDA (0.5% w/v in acetone) was diluted with distilled water to create a fresh 0.01% w/v FDA working solution kept in the dark at 4°C. Grapevine cell suspensions were aliquoted in 2 mL cell culture package on Poly-Prep chromatography columns (BioRad) and then diluted 1/10 with PBS (2.7 mM KCl, 137 mM NaCl, 1.8 mM KH_2_PO_4_, 4.0 mM Na_2_HPO_4_). One hundred µL of this solution were mixed by gentle stirring with 0.01% w/v FDA in a quartz cuvette. A spectrophotofluorimeter (Perkin Elmer, UK) equipped with a stirrer was employed. Excitation and emission wavelengths were selected at 493 and 510 nm, respectively. The increase in fluorescence was recorded over a 120 s time period. The slope of the fluorescence increase (between 60 to 90 s) was calculated for each cellular suspension to determine the correlation between cell viability and the velocity of FDA conversion. A standard cell viability curve was set up. Dead cells were prepared by boiling viable cells. Aliquots of control dead cells were added in different quantities to healthy viable cells to obtain ranges between 0 and 100%.

### Statistical analysis

Data are presented as mean and standard deviation of the biological and technical replicates. Differences were tested by unpaired, two-tailed T test. Significant differences by p<5% or p<1% are indicated by one or two asterisks, respectively.

## Results

### Physiological and molecular characterization of leaf senescence

**Figure 1:**
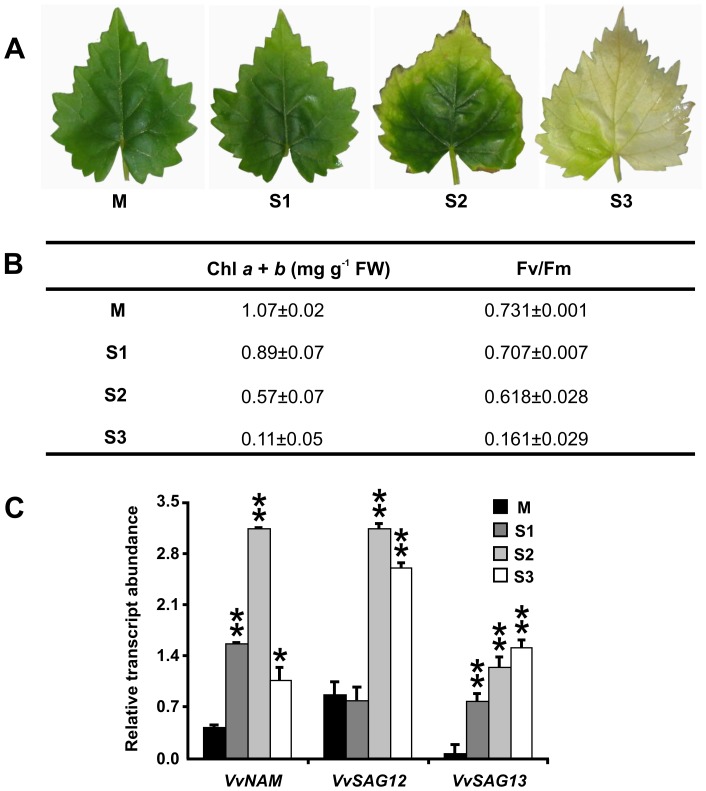
Leaf senescence in grapevine plants. (A) Stages of leaf senescence: M (mature green leaf), S1, S2 and S3 (senescent leaves). (B) Photosynthetic parameters: content of chlorophyll *a* and *b*, efficiency of photosystem II (Fv/Fm). (C) Expression analysis of *VvNAM*, *VvSAG12* and *VvSAG13* at different leaf senescence stages (M, S1, S2 and S3). The data are reported as mean ± S.D of values of transcript abundance relative to the housekeeping genes, from 20 replicates. Asterisks indicate values that are statistically different from those of M stage by Student's *t* test (** *P*<0.01; * *P*<0.05).

Leaf senescence was assessed for three months in grapevine plants grown *in vitro*. Following a common classification for senescent leaves [28], [29], we identified four different stages, based on the progressive chlorophyll loss. Expanded green leaves (M) had the maximum chlorophyll content, whereas in the subsequent senescent stages S1 (30 day-old), S2 (60 day-old) and S3 (90 day-old) the chlorophyll loss was 17, 47 and 90%, respectively (Figure 1A, B). Chlorophyll degradation was accompanied by a decrease in the photosynthetic capacity, another parameter useful in identifying senescence stages [30]. Leaves remain active during the first stages of senescence, as evidenced by the stable values of photosynthetic efficiency (Fv/Fm) of photosystem II (PSII) until stage S2. In S3, photosynthetic efficiency dropped, in agreement with the low chlorophyll content measured in this stage (Figure 1B).

For the molecular characterization, the transcriptional profiles of known molecular markers of senescence, namely *VvNAM*, *VvSAG12* and *VvSAG13* genes [31], normalised against the expression of the housekeeping actin gene, were analysed. During senescence, *VvNAM* and *VvSAG12* expression levels increased from stage M to S2, and then declined. The transcript level of *VvSAG13*, instead, constantly increased until stage S3 (Figure 1C).

### Analyses of mitochondrial morphology and dynamics during leaf senescence

**Figure 2:**
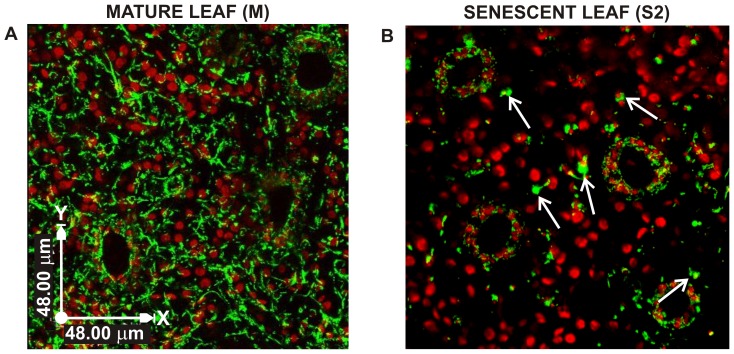
Mitochondrial morphology and distribution in mature and senescent leaves of grapevine plants grown in vitro, expressing β-GFP. (A) Bi-dimensional GFP-fluorescence image resulted from a projection of laser scanning confocal image sequences, showing long branched structures formed by mitochondria in a mature leaf. (B) Bi-dimensional projection image of senescent leaf in S2 senescence stage showing enlarged, round shape mitochondria (see arrows). Scale bar  = 48 µm.

**Figure 3:**
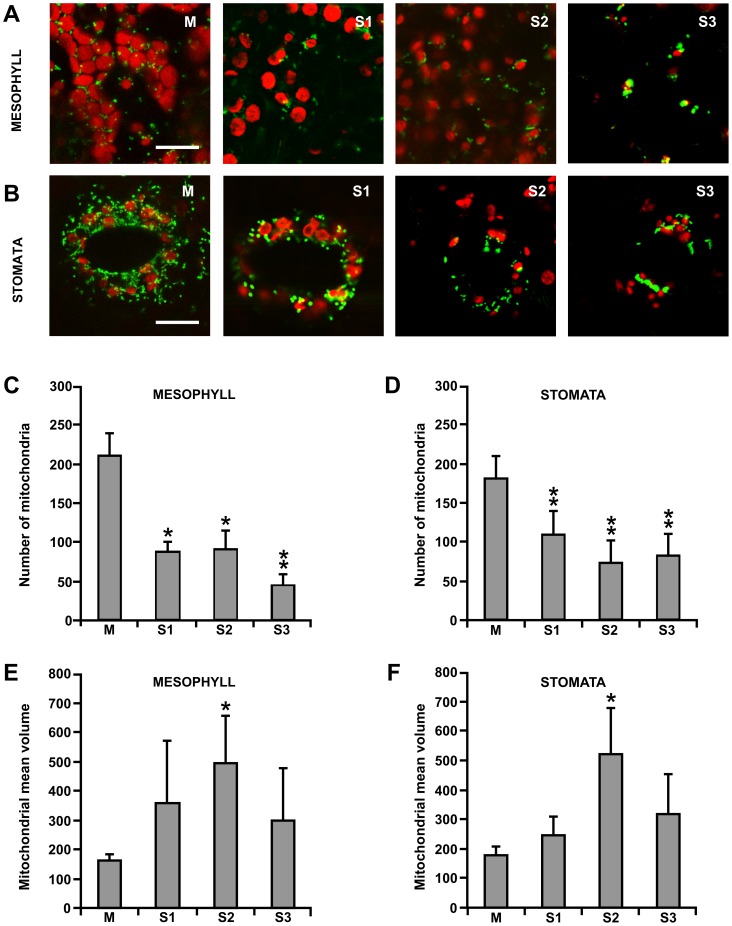
Visualization by confocal laser microscopy of mitochondria in leaves of grapevine plants, expressing β-GFP during senescence. Confocal analyses in mesophyll (A) and stomata (B) cells at different senescence phases: M (mature green leaf), S1, S2 and S3 (senescent leaves). Scale bar  = 10 µm. The number and volume of mitochondria were quantified in mesophyll (C, E) and stomata (D, F) leaf cells at different senescence stages. Asterisks indicate values that are statistically different from those of M stage by Student's *t* test (** *P*<0.01; * *P*<0.05), performed on 10 replicates.

Mitochondria were visualized by stable expression of the fluorescent reporter GFP carrying the sequence tag for mitochondrial localization (β-GFP) (see Figure S1 for details on transformation procedures). At stage M, mitochondria appeared abundant and inter-connected in long, branched structures, homogeneously distributed throughout the cytoplasm (Figure 2A). In senescent leaves (S2 stage), mitochondria were scarce, isolated and had a different morphology, characterized by a large, round shape (Figure 2B). Since senescence seems to follow different routes in mesophyll and stomata cells [32], [33], [3], we analysed mitochondria morphology in details in these cell types (Figure 3A,B). The decrease in the mitochondria number during senescence was more pronounced in mesophyll cells (decrease of 25% at stage S3 as compared to M, Figure 3C) than in stomata (decrease of 50%, at stage S3 as compared to M, Figure 3D). In both cell types, the mean volume of mitochondria increased at S2 and then decreased again during S3 (Figure 3E,F). If we consider the total mitochondrial volume, measured in the different stages of leaf development, we observed that is was substantially unchanged up to the S2, while in the S3 stage the total volume and the total number of mitochondria drastically decreased.

**Figure 4:**
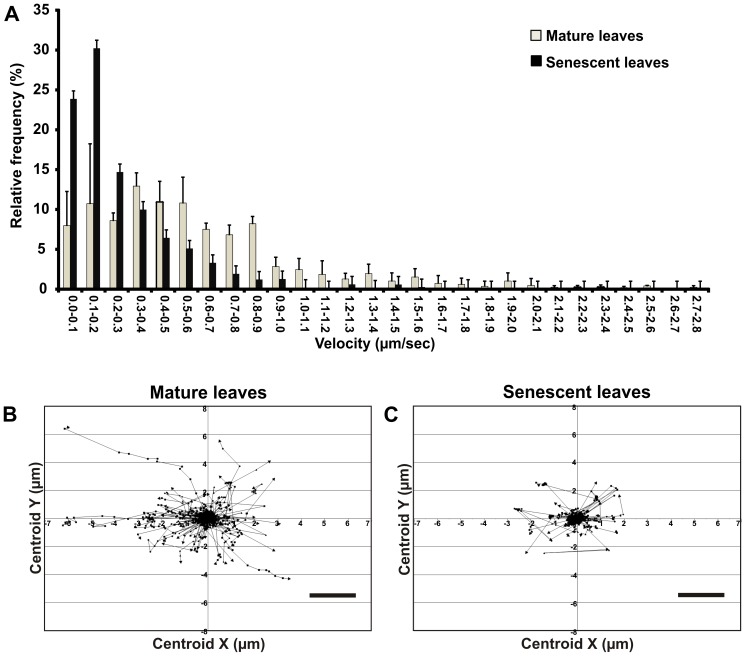
Analysis of mitochondria dynamics in leaves of grapevine plants. Time-laps movies were acquired, and mitochondria velocity (A) and tracking (B, C) were analysed by Volocity® 6.0 software. (A) Relative frequency of mitochondrial velocities in grapevine leaf epidermal cells of mature and S2 senescent leaves. Values represent mean ± S.D of at least 100 oranelles from three independent replicates. (B, C) Tracking of mitochondria, in mature (B) and S2 senescent (C) leaves. In B and C dots represent position of mitochondria in planar space. Initial position is artificially centralized. Data were acquired every 1.6 seconds and connecting black lines show the shortest path possible. Scale bar  = 2 µm.

In order to determine whether mitochondrial movement was different in mature and senescent epidermal leaf tissues, dynamics of mitochondria was analysed in several time-lapse movies (Movie S1; Movie S2). Figure 4 (A) shows the distribution of mitochondria velocity (µm/sec), measured in both mature and senescent leaves. It is noteworthy that the majority of senescent leaf mitochondria were slow (54% with ≤0.2 µm/sec), while a broad distribution of mitochondria velocity was observed in mature leaves (in mature leaves the average and median of mitochondria velocity were 0,64 and 0.51 µm/sec respectively, while in senescent leaves average and median were 0,32 and 0.22 µm/sec respectively). Figure 4 (B,C) describes the movement patterns of mitochondria populations in mature (B) and senescent (C) leaves, reported as displacement rates. Also in this case, two different behaviours are evident, with mitochondria from senescent leaves less dynamic than those from mature leaves.

### Effects of high levels of the cytokinin on leaf senescence

**Figure 5:**
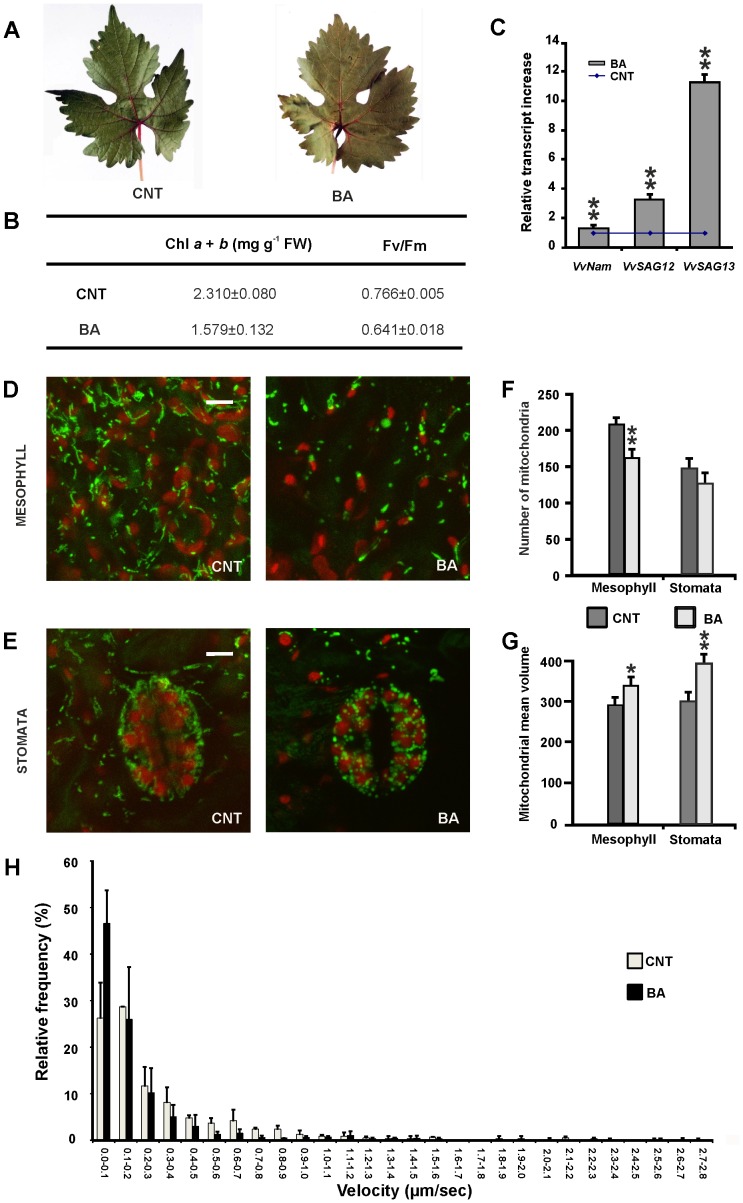
Cytokinin-induced senescence in leaves of grapevine stem cuttings. (A) Untreated (CNT) or treated with 100 µM BA leaves, analysed 12 days after treatment. (B) Photosynthetic parameters: content of chlorophyll *a* and *b*, efficiency of photosystem II (Fv/Fm). Values represent mean ± S.D of three independent replicates (C) Gene expression analysis in stem cuttings treated with 100 µM BA. Data are shown as fold increase of *VvNAM*, *VvSAG12* and *VvSAG13* transcript abundance in treated stem cuttings as compared to untreated stem cuttings (set as 1, blue line). Values represent mean ± S.D of 20 replicates. Asterisks indicate values that are statistically different from those of untreated stem cuttings by Student's *t* test (** *P*<0.01). (D, E) Confocal microscope analyses of mitochondrial morphology after cytokinin treatment in stomata and mesophyll cells. Scale bar  = 5 µM. The number (F) and volume (G) of mitochondria were quantified in mesophyll and stomata s. Asterisks indicate values that are statistically different from those of untreated samples by Student's *t* test (** *P*<0.01; * *P*<0.05). (H) Relative frequency of mitochondrial velocities in grapevine leaf epidermal cells in control and cytokinin treated (100 µM BA for 12 days, see text for details) leaf tissue of stem cuttings. Values represent mean ± S.D of at least 100 organelles from three independent replicates.

Senescence is a slow process, under the influence of several endogenous and environmental stimuli. Under certain conditions, young and mature leaves can be induced to undergo a process of accelerated senescence and PCD. We had previously reported that high concentrations of cytokinins are able to induce a senescence-like process both in plants and cell cultures [9], [10]. In order to check whether mitochondria alterations observed during spontaneous senescence were also detectable when senescence was artificially induced, grapevine stem cuttings treated with high levels of cytokinins were analysed. Leaf treatment with 100 µM BA affected chlorophyll content (32% loss) and photosynthetic efficiency (16% decrease of Fv/Fm) as compared to untreated leaves (Figure 5A,B), and induced a strong increase of the senescence markers *VvSAG12* and *VvSAG13* expression (Figure 5C). Altogether, these results show that high concentrations of the cytokinin BA induced a senescence-like process in grapevine leaves. Mitochondria abundance and morphology were then analysed both in stomata and mesophyll cells (Figure 5D–G). As expected, mitochondria were abundant, elongated and forming dynamic branched structures in untreated leaves (Figure 5D,E; Movie S3). Cytokinin treatment resulted in dramatic alterations in mitochondrial morphology and dynamics. In presence of 100 µM BA, the branched structures disappeared, and mitochondria appeared enlarged and round-shaped (Figure 5D,E,G). In parallel, cytokinin treatment caused a significant reduction in the mitochondrial number in the mesophyll cells (Fig 5F). Mitochondria dynamics was also analysed in leaf epidermal cells upon cytokinin treatment and a strong delay in velocity was detected and measured as shown in Figure 5H and Movie S4 (the average and median of mitochondria velocity in untreated leaves were 0.34 and 0.18 µm/sec, respectively; while in senescent leaves were 0.22 and 0.10 µm/sec, respectively).

### Analyses of mitochondrial morphology in suspension cell cultures

**Figure 6:**
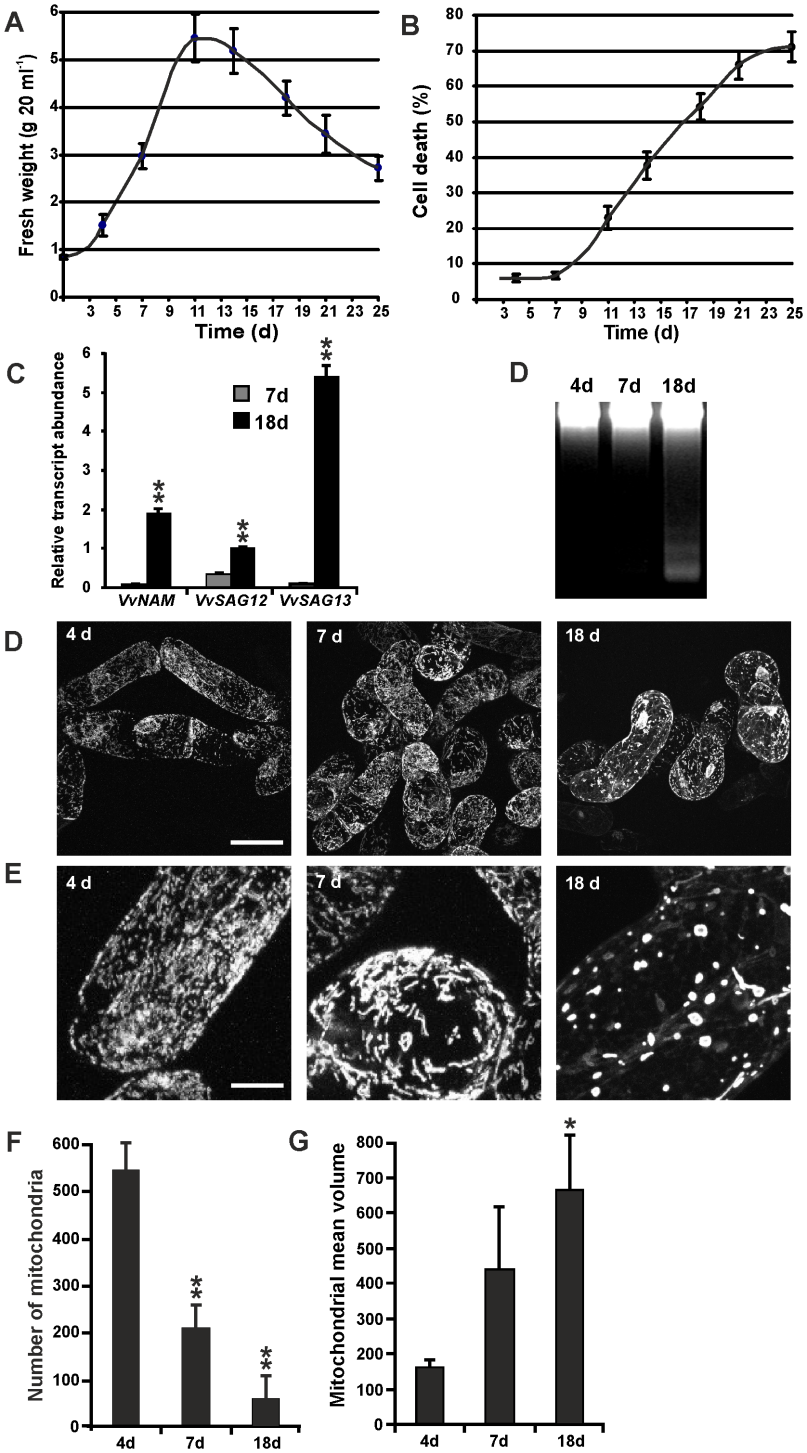
Alterations of mitochondrial morphology during senescence in grapevine suspension cell cultures. (A) Cell growth shown as fresh weight of cultured cells. (B) Cell death estimated by FDA staining. (C) Expression analysis of *VvNAM*, *VvSAG12* and *VvSAG13* during exponential growth stage (7d) and senescence (18d). Values represent mean ± S.D. of 20 replicates. Asterisks indicate values that are statistically different from those of 7d stage by Student's *t* test (** *P*<0.01). (D) Agarose gel analysis of DNA extracted from 4-, 7-, 18-day-old cultures. (E) Confocal microscopy analyses of mitochondrial morphology in senescent suspension cell cultures at different times after culture initiation (4, 7 and 18 days). Scale bar  = 20 µM. (F) Enlarged picture of mitochondria: 4-day-old culture with numerous small mitochondria; 7-day-old culture with elongated mitochondria; 18-day-old culture with a reduced number of large mitochondria (giant spherical and ring-shaped). Scale bar  = 5 µm. The number (G) and volume (H) of mitochondria were quantified in 4, 7 and 18 days old cells. Asterisks indicate values that are statistically different from those of 4d stage by Student's *t* test (** *P*<0.01; * *P*<0.05), performed on nine replicates.

Senescence is a complex phenomenon, involving nutrient recycling between separate plant organs. Even within a single leaf, cell types are affected differently during senescence, as shown for stomata and mesophyll cells. In order to reduce this source of variability, it is convenient to have a study system with a reduced complexity. Cell cultures present several advantages as model system, since they are homogeneous, fast-growing and chemical treatments are easily applied. In order to test whether the changes associated with leaf senescence could be detectable in cell cultures, we established and characterized a cell line from grapevine leaves. Suspension cell cultures actively proliferated during the first 11 days after a subculture (Figure 6A) and then progressively declined and died due to nutrient depletion (Figure 6B). We previously reported that starvation triggers a senescence-like PCD process in Arabidopsis cell cultures [10], [11]. In order to test whether grapevine cell cultures had features similar to leaf senescence, we evaluated the expression pattern of *VvNAM*, *VvSAG12* and *VvSAG13*. The senescence gene markers were expressed at low levels in cells at mid-exponential growth phase (7 days after culture initiation), but strongly increased in the decline phase (Figure 6C; 18 days), confirming that cell cultures are a suitable model to study certain aspects of leaf senescence.

In addition, the cell DNA integrity was measured as a marker of PCD. As reported in Fig. 6D, the typical DNA laddering pattern was observed in DNA from 18 day old cells, when the percentage of cell death was 55%. This result is in agreement with previous data reported in Arabidopsis cultured cells, where it has been shown that DNA laddering was detectable only when the level of cell death reached 40% [11].

Mitochondrial morphology was analysed at different times of the sub-culture cycle through tetramethylrhodamine methyl ester (TMRM) staining (Figure 6E,F). At the initial phase of the growth curve (4 days after culture initiation), mitochondria were punctiform, abundant and homogeneously distributed into the cytoplasm (Figure 6E,F; 4 days). At the mid-exponential phase of growth (7 days), elongated and branched mitochondrial structures were detected (Figure 6E,F). At 18 days, in advanced senescence phase (55% of cells were already dead, Figure 6B), mitochondria were scarce and appeared as giant spherical or ring-shaped organelles (Figure 6F; 18 days). Quantification of mitochondria number and volume in senescing cells showed a ten-fold reduction of mitochondria number while the volume increased more than four-fold (Figure 6G, H).

## Discussion

Senescence is a fundamental phase in plant organ development and, as such, has strong influence on crop quality, productivity and fruit shelf-life. Understanding the molecular mechanisms underlying senescence could provide new tools for enhanced crop management and breeding. Control of senescence might result in increased photosynthetic lifetime of leaves and augmented biomass. For fruit crops, senescence affects source-sink relationship through nutrient recycling. Moreover, fruit ripening itself can be considered a type of senescence. *V. vinifera*, a perennial fruit crop with a full seasonal leaf senescence syndrome, and a sequenced, annotated genome, represents therefore an optimal model species for studying senescence.

In the present work, we first characterized leaf senescence in grapevine by physiological (chlorophyll content, photosynthetic efficiency) and molecular (pattern of expression of senescence marker genes) analyses. We then focused on the alterations of mitochondria morphology and dynamics during senescence. Transgenic grapevine plants harbouring mitochondria-targeted GFP represent a new, useful tool for studying the role of these organelles in woody plants. Stable expression lines avoided the artefacts induced by agroinfiltration procedure, due to wounding and heterogeneous transformation events. Moreover, it allowed the long-term assessment of mitochondrial pattern evolution during the slow process of spontaneous leaf senescence.

Our analyses revealed that mitochondria underwent dramatic changes during leaf senescence. In mature leaves, mitochondria appeared as long, branched structures formed by inter-connected mitochondria. These structures gradually evolved, along senescence, toward enlarged, round-shaped mitochondria, and in the meantime the total number of organelles significantly decreased. These alterations were particularly evident in the S2 senescence phase, when leaves had lost about 50% of their chlorophyll content and the expression of senescence marker genes, coding for proteins involved in nutrient recycling [31], highly increased.

Basing on our previous studies, we also show that treatment of grapevine cuttings with high levels of the cytokinin BA induced an accelerated process of leaf senescence and affected mitochondrial morphology similarly to spontaneous senescence. This experimental approach provides therefore a rapid, reproducible system in which senescence is induced in a short and defined period of time. It is worth mentioning that the induction of senescence by cytokinins was stage dependent. In fact, BA was effective on expanding leaves but not on mature, completely expanded ones (results non reported), suggesting that in leaves, as previously shown in cell cultures [9], a sort of cell/tissue competence dependent on the developmental stage is required.

An interesting observation of the present work is that mitochondria behave slightly different in stomata and mesophyll cells during senescence. In particular, mitochondria appeared more structured in stomata than in mesophyll cells (Figure 2 and 3), possibly because in stomata, senescence is not as orderly paced as in mesophyll cells [33]. The apparent delay in the onset of senescence in guard cells might be instrumental in ensuring functional stomata and gas exchange until the senescence process undergoing in the other leaves cells is completed.

Our observations indicate that timing and progression of senescence differ among cell types in leaves. This heterogeneity, and the experimental difficulties that characterize the studies on perennial woody species, make cell cultures an attractive system. Being homogeneous, fast growing, easy to handle and readily accessible to treatments, cell cultures might represent an ideal study system. Previous observations from our laboratory had shown that mitochondria undergo modifications in their morphology along the growth curve of a cell culture of *M. truncatula* [8]. This prompted us to look for a correlation between leaf senescence and nutrient depletion in cell cultures of grapevine. Analysis of the expression pattern of three senescence marker genes, detected in the final phase of cell growth curve, confirmed that senescence of cell cultures mimics leaf senescence also at a molecular level. Similar alterations in mitochondrial morphology and dynamics were observed in senescing leaves and cell cultures. We foresee that cell cultures can be used as a first, convenient study system, to test the effect of different treatments on senescence.

An important finding of this study is that spontaneous as well cytokinin-induced leaf senescence were accompanied by a general and strong decrease in mitochondria motility, about 40–60% lower than mature and untreated stem cuttings leaves. This effect might depend on the progressive degradation of the cytoskeleton [34]. It is currently unclear whether mitochondria motility controls, or is controlled by, the physiological state of cells. Recent reports suggest that mitochondria motility is correlated to calcium signalling, both in animal [35] and plants [36]. The use of new tools for measuring *in vivo* mitochondrial calcium concentration and dynamics, such as genetically encoded sensors [37], might contribute to clarify the role of calcium in the regulation of the senescence process. A growing number of genetically encoded sensors for a variety of ions and metabolites have been successfully employed in mitochondria [38]. Since senescence in plants involves nitrogen recycling, the recently developed activity sensors for ammonium transport might provide an interesting tool [39].

In conclusion, here we report alterations in mitochondria number, morphology and dynamics during spontaneous and cytokinin-induced senescence of grapevine leaves and cell cultures. Long, branched mitochondria characterizing mature and early senescent leaves progress into enlarged, less motile mitochondria in the late senescent stages. In this context, mitochondria morphologies represent useful markers to identify different physiological and developmental stages of senescence.

## Supporting Information

Figure S1 Transgenic grapevine embryogenic cell lines stably expressing β-GFP targeted to mitochondria. (A) Epi-fluorescence images of embryogenic callus: starting material for transformation procedure (t_0_); 10 days after transformation (t_1_); 3 months after transformation (t_2_; arrows indicate transformed globular embryos); 4 months after transformation (t_3_; arrows indicate torpedo embryos). (B) Epi-fluorescence (1, 2) and confocal (3, 4) microscopy images of heart-stage transgenic embryos at different magnifications. (C) Epi-fluorescence microscopy images of secondary embryogenesis from primary transformants and regeneration of an adult plant: (1) Primary transformed embryo; (2) Secondary somatic embryos regenerated from hypocotyl surrounding tissues; (3) Transformed root and (4) transformed leaf of plants obtained from the secondary embryos.
(TIF)

Movie S1 Movement of β-GFP-labeled mitochondria in leaf at mature M stage. The leaf was analysed by means of confocal laser scanning microscope. The movie was recorded at 1 frame per 1.6 seconds. Playback is at 7.5 frames/second (12x normal speed), so the movie represents 48 seconds of real time.
(AVI)

Movie S2 Movement of β-GFP-labeled mitochondria in leaf at the senescent S2 stage. The movie was recorded at 1 frame per 1.6 seconds and is playing at 7.5 frames/second, representing 48 seconds of real time (12x normal speed).
(AVI)

Movie S3 Movement of β-GFP-labeled mitochondria in control leaf of hydroponic grown plant. The movie was recorded at 1 frame per 1.6 seconds and is playing at 7.5 frames/second, representing 48 seconds of real time (12x normal speed).
(AVI)

Movie S4 Movement of β-GFP-labeled mitochondria in leaf of hydroponic grown grapevine plants treated with 100 µM BA. The movie was recorded at 1 frame per 1.6 seconds and is playing at 7.5 frames/second, representing 48 seconds of real time (12x normal speed).
(AVI)

## References

[1] Yoshida S (2003) Molecular regulation of leaf senescence. Curr Opin Plant Biol 6: 79–84. 12495755 10.1016/s1369526602000092

[2] Breeze E, Harrison E, McHattie S, Hughes L, Hickman R, et al (2011) High-resolution temporal profiling of transcripts during Arabidopsis leaf senescence reveals a distinct chronology of processes and regulation. Plant Cell 23: 873–894. 21447789 10.1105/tpc.111.083345 PMC3082270

[3] Keech O, Pesquet E, Ahad A, Askne A, Nordvall D, et al (2007) The different fates of mitochondria and chloroplasts during dark-induced senescence in Arabidopsis leaves. Plant Cell Environ 30: 1523–1534. 17986154 10.1111/j.1365-3040.2007.01724.x

[4] Siedow JN, Day DA (2000) Respiration and photorespiration. In: Biochemistry and Molecular Biology of Plants (Buchanan BB, Gruissem W, Jones RL eds), pp. 676–728. American Society of Plant Physiologists.

[5] Reape TJ, McCabe PF (2008) Apoptotic-like programmed cell death in plants. New Phytol 180: 13–26. 18631291 10.1111/j.1469-8137.2008.02549.x

[6] Logan DC (2010) The dynamic plant chondriome. Semin Cell Dev Biol 21: 550–557. 20044013 10.1016/j.semcdb.2009.12.010

[7] Scott I, Logan DC (2008) Mitochondrial morphology transition is an early indicator of subsequent cell death in Arabidopsis. New Phytol 177: 90–101. 17986180 10.1111/j.1469-8137.2007.02255.x

[8] Zottini M, Barizza E, Bastianelli F, Carimi F, Lo Schiavo F (2006) Growth and senescence of *Medicago truncatula* cultured cells are associated with characteristic mitochondrial morphology. New Phytol 172: 239–247. 16995912 10.1111/j.1469-8137.2006.01830.x

[9] Carimi F, Zottini M, Formentin E, Terzi M, Lo Schiavo F (2003) Cytokinins: New apoptotic inducers in plants. Planta 216: 413–421. 12520332 10.1007/s00425-002-0862-x

[10] Carimi F, Terzi M, De Michele R, Zottini M, Lo Schiavo F (2004) High levels of cytokinin BAP induce PCD by accelerating senescence. Plant Sci 166: 963–969.

[11] Carimi F, Zottini M, Costa A, Cattelan I, De Michele, et al (2005a) NO signalling in cytokinin-induced programmed cell death. Plant Cell Environ 28: 1171–1178.

[12] Vlčková A, Špundová M, Kotabová E, Novotný R, Doležal K, et al (2006) Protective cytokinin action switches to damaging during senescence of detached wheat leaves in continuous light. Physiol Plant 126: 257–267.

[13] Piotrowska A, Czerpak R (2009) Cellular response of light/dark-grown green alga *Chlorella vulgaris Beijerinck* (Chlorophyceae) to exogenous adenine- and phenylurea-type cytokinins. Acta Physiol Plant 31: 573–585.

[14] Jaillon O, Aury JM, Noel B, Policriti A, Clepet C, et al (2007) The grapevine genome sequence suggests ancestral hexaploidization in major angiosperm phyla. Nature 449: 463–467. 17721507 10.1038/nature06148

[15] Velasco R, Zharkikh A, Troggio M, Cartwright DA, Cestaro A, et al (2007) High quality draft consensus sequence of the genome of a heterozygous grapevine variety. PLOS ONE 2: e1326. 18094749 10.1371/journal.pone.0001326 PMC2147077

[16] Goremykin VV, Salamini F, Velasco R, Viola R (2009) Mitochondrial DNA of *Vitis vinifera* and the issue of rampant horizontal gene transfer. Mol Biol Evol 26: 99–110. 18922764 10.1093/molbev/msn226

[17] Zhao R, Dielen V, Kinet JM, Bountry M (2000) Cosuppression of a plasma membrane H+-ATPase isoform impairs sucrose translocation, stomatal opening, plant growth, and male fertility. Plant Cell 12: 535–546. 10760242 10.1105/tpc.12.4.535 PMC139851

[18] Duby G, Oufattole M, Boutry M (2001) Hydrophobic residues within the predicted N-terminal amphiphilic alpha-helix of a plant mitochondrial targeting presequence play a major role in *in vivo* import. Plant J 27: 539–549. 11576437 10.1046/j.1365-313x.2001.01098.x

[19] Zottini M, Barizza E, Costa A, Formentin E, Ruberti C, et al (2008) Agroinfiltration of grapevine leaves for fast transient assays of gene expression and for long-term production of stable transformed cells. Plant Cell Rep 27: 845–853. 18256839 10.1007/s00299-008-0510-4

[20] Sambrook J, Fritsch EF, Maniatis T (1989) Molecular Cloning: A Laboratory Manual, 2nd edn. Cold Spring Harbor: Cold Spring Harbor Laboratory Press.

[21] Carimi F, Barizza E, Gardiman M, Lo Schiavo F (2005b) Somatic embryogenesis from stigmas and styles of grapevine. In Vitro Cell Dev Biol, Plant 41: 249–252.

[22] Nitsch JP, Nitsch C (1969) Haploid plants from pollen grains. Science 163: 85–87. 17780179 10.1126/science.163.3862.85

[23] Murashige T, Skoog F (1962) A revised medium for rapid growth and bioassays with tobacco tissue cultures. Physiol Plantarum 15: 473–497.

[24] Gamborg OL, Miller RA, Ojima K (1968) Nutrient requirements of suspension cultures of soybean root cells. Exp Cell Res 50: 151–158. 5650857 10.1016/0014-4827(68)90403-5

[25] Porra RJ, Thompson WA, Kriedemann PF (1989) Determination of accurate extinction coefficients and simultaneous equations for assaying chlorophyll *a* and *b* extracted with four different solvents: verification of the concentration of chlorophyll standards by atomic absorption spectrophotometry. Biochim Biophys Acta 975: 384–394.

[26] Demmig-Adams B, Adams WW III, Barker DH, Logan BA, Bowling DR, et al (1996) Using chlorophyll fluorescence to assess the fraction of absorbed light allocated to thermal dissipation of excess excitation. Physiol Plant 98: 253–264.

[27] Amano T, Hirasawa K, O'Donohue MJ, Pernolle JC, Shioi Y (2003) A versatile assay for the accurate, time-resolved determination of cellular viability. Anal Biochem 314: 555–565. 10.1016/s0003-2697(02)00653-x 12633596

[28] De Michele R, Formentin E, Todesco M, Toppo S, Carimi F, et al (2009a) Transcriptome analysis of *Medicago truncatula* leaf senescence: similarities and differences in metabolic and transcriptional regulations as compared with Arabidopsis, nodule senescence and nitric oxide signaling. New Phytol 181: 563–575. 19021865 10.1111/j.1469-8137.2008.02684.x

[29] De Michele R, Formentin E, Lo Schiavo F (2009b) Legume leaf senescence: a transcriptional analysis. Plant Sign Behav 4(4): 1–2. 10.4161/psb.4.4.8116 PMC2664495 19794851

[30] Lim HG, Kim HJ, Nam HG (2007) Leaf senescence. Annual Rev Plant Biol 58: 115–136. 17177638 10.1146/annurev.arplant.57.032905.105316

[31] Espinoza C, Medina C, Somerville S, Arce-Johnson P (2007) Senescence-associated genes induced during compatible viral interactions with grapevine and Arabidopsis. J Exp Bot 58: 3197–3212. 17761729 10.1093/jxb/erm165

[32] Zeiger E, Schwartz A (1982) Longevity of guard cell chloroplasts in falling leaves: implication for stomatal function and cellular aging. Science 218: 680–682. 17791588 10.1126/science.218.4573.680

[33] Ozuna R, Year R, Ortega K, Tallman G (1985) Analysis of guard cell viability and action in senescing leaves of *Nicotiana glauca* (graham), tree tobacco. Plant Physiol 79: 7–10. 16664404 10.1104/pp.79.1.7 PMC1074820

[34] Keech O, Pesquet E, Gutierrez L, Ahad A, Bellini C, et al (2010) Leaf senescence is accompanied by an early disruption of the microtubule network in Arabidopsis. Plant Physiol 154: 1710–1720. 20966154 10.1104/pp.110.163402 PMC2996031

[35] Chang KT, Niescier RF, Minb K-T (2011) Mitochondrial matrix Ca2+ as an intrinsic signal regulating mitochondrial motility in axons. PNAS 108: 15456–15461. 21876166 10.1073/pnas.1106862108 PMC3174631

[36] Yamaoka S, Leaver CJ (2008) EMB2473/MIRO1, an Arabidopsis Miro GTPase, is required for embryogenesis and influences mitochondrial morphology in Pollen. Plant Cell 20: 589–601. 18344283 10.1105/tpc.107.055756 PMC2329936

[37] Loro G, Drago I, Pozzan T, Lo Schiavo F, Zottini M, et al (2012) Targeting of Cameleons to various subcellular compartments reveals a strict cytoplasmic/mitochondrial Ca(2)(+) handling relationship in plant cells. Plant J 71: 1–13. 22372377 10.1111/j.1365-313X.2012.04968.x

[38] De Michele R, Carimi F, Frommer W (2014) Mitochondrial biosensors. Inter J Biochem Cell Biol 48: 39–44. 10.1016/j.biocel.2013.12.014 24397954

[39] De Michele R, Ast C, Loqué D, Ho C-H, Andrade SL, et al (2013) Fluorescent sensors reporting the activity of ammonium transceptors in live cells. eLife. 2: e01029 doi:10.7554/eLife.01029 10.7554/eLife.00800 PMC3699834 23840931

