# Supplementary figures and images for: Mitochondria Change Dynamics and Morphology during Grapevine Leaf Senescence
Source: PLoS One. 2014 Jul 10;9(7):e102012. doi: 10.1371/journal.pone.0102012 (PMC4092070; doi:10.1371/journal.pone.0102012)

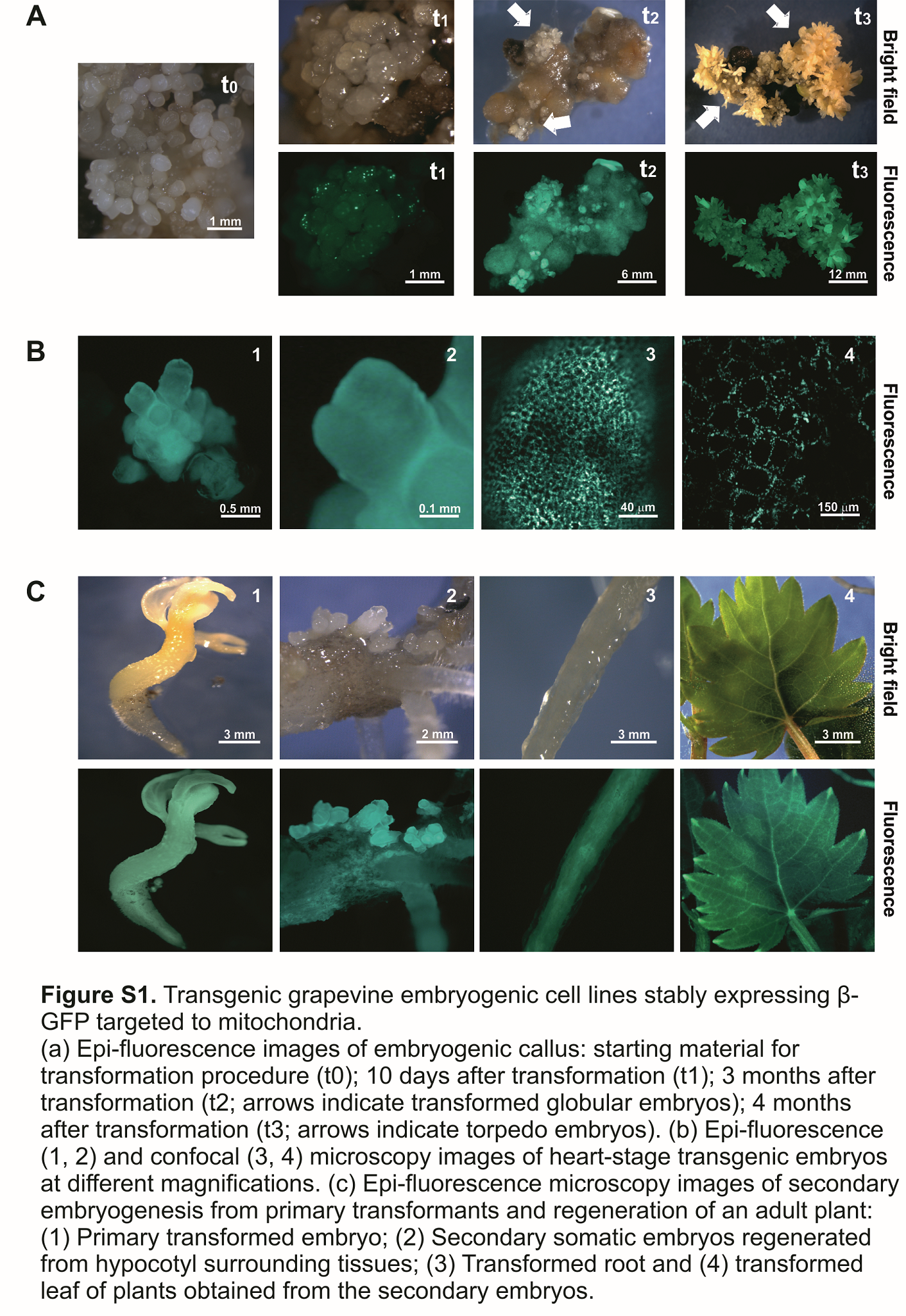

Supplement: Figure S1 — Transgenic grapevine embryogenic cell lines stably expressing β-GFP targeted to mitochondria. (A) Epi-fluorescence images of embryogenic callus: starting material for transformation procedure (t0); 10 days after transformation (t1); 3 months after transformation (t2; arrows indicate transformed globular embryos); 4 months after transformation (t3; arrows indicate torpedo embryos). (B) Epi-fluorescence (1, 2) and confocal (3, 4) microscopy images of heart-stage transgenic embryos at different magnifications. (C) Epi-fluorescence microscopy images of secondary embryogenesis from primary transformants and regeneration of an adult plant: (1) Primary transformed embryo; (2) Secondary somatic embryos regenerated from hypocotyl surrounding tissues; (3) Transformed root and (4) transformed leaf of plants obtained from the secondary embryos. (TIF) [file pone.0102012.s001.tif]
